# The evolution of sex-specific immune defences

**DOI:** 10.1098/rspb.2010.0188

**Published:** 2010-03-24

**Authors:** Olivier Restif, William Amos

**Affiliations:** 1 Department of Veterinary Medicine, University of Cambridge, Madingley Road, Cambridge CB3 0ES, UK; 2 Department of Zoology, University of Cambridge, Downing Street, Cambridge CB2 3EJ, UK

**Keywords:** adaptive dynamics, evolutionarily stable strategies, pathogens, coevolution, sexual reproduction, immunocompetence

## Abstract

Why do males and females often differ in their ability to cope with infection? Beyond physiological mechanisms, it has recently been proposed that life-history theory could explain immune differences from an adaptive point of view in relation to sex-specific reproductive strategies. However, a point often overlooked is that the benefits of immunity, and possibly the costs, depend not only on the host genotype but also on the presence and the phenotype of pathogens. To address this issue we developed an adaptive dynamic model that includes host–pathogen population dynamics and host sexual reproduction. Our model predicts that, although different reproductive strategies, following Bateman's principle, are not enough to select for different levels of immunity, males and females respond differently to further changes in the characteristics of either sex. For example, if males are more exposed to infection than females (e.g. for behavioural reasons), it is possible to see them evolve lower immunocompetence than females. This and other counterintuitive results highlight the importance of ecological feedbacks in the evolution of immune defences. While this study focuses on sex-specific natural selection, it could easily be extended to include sexual selection and thus help to understand the interplay between the two processes.

## 1. Introduction

Sex affects a wide range of traits in animals, from anatomy to physiology and behaviour, often but not exclusively linked to reproduction. Such sexual dimorphism can evolve in response to a variety of ecological factors combined with sexual selection (Shine 1989). Parasites and pathogens have been shown to play a significant role in this process through selection on immune defences (Folstad & Karter 1992; Moore & Wilson 2002). The relationship between immune defences and reproductive success has been extensively studied (Sorci & Clobert 1995; Nordling *et al*. 1998; Cornet *et al*. 2009), as has sexual dimorphism in immunity, both in the ecological (Møller & Sorci 1998; Caillaud *et al*. 2006) and the biomedical literature (May 2007; Strachan *et al*. 2008; Meier *et al*. 2009). Reported differences include males being more exposed to infection risk than females (Semple *et al*. 2002), being less able than females to deal with infection (Lindsey & Altizer 2009), shedding more viral particles (Lin *et al*. 2006) or suffering more severe symptoms (Cernetich *et al*. 2006), while in some systems females exhibit higher susceptibility to infection (Guilbault *et al*. 2002). Clinical and experimental studies have started to identify proximal causes for sex differences in immunity (Klein 2005; Pasche *et al*. 2005)—in particular the way sex hormones can modulate immune responses—but the evolutionary context of these differences has been largely ignored (Zuk 1990, 2008).

Immunity impacts either directly or indirectly on both survival and reproductive success. Consequently, wherever the sexes differ in their reproductive strategies, natural selection may operate to promote sex differences in immunity. As Zuk (1990, 2008) states, given physiological constraints on the level of investment in immunity, intrinsic differences in reproductive strategies between males and females should determine their respective immunocompetence. By implication, life-history theory should be able to predict how interspecific variations in reproductive traits may correlate with sex bias in immune defences. A recent model was proposed by Stoehr & Kokko (2006) to investigate optimal allocation of resources between immunity, survival and reproduction in males and females, under varying levels of sexual selection by females. This model can be seen as a first attempt to formalize the ideas of Zuk (1990), but we argue that it lacks three essential elements: a genetic framework, ecological dynamics and evolutionary dynamics. First, a genetic framework is needed because, ignoring the Y/Z chromosomes, males and females carry the same genes and these recombine across the sexes at every generation. Second, ecological dynamics, in particular host–parasite dynamics, are crucial to determine the adaptive benefits of immunity as well as reproductive success (Roy & Kirchner 2000; Restif & Koella 2003). Last, the genetic and ecological components must be incorporated into a proper evolutionary framework where fitness is not determined in isolation, but is instead directly related to the effective reproductive success of a given genotype in a polymorphic population.

There are many ways in which the above programme can be implemented, and the model we propose attempts to incorporate all these elements into a reasonably simple and tractable framework. The general motivation of the present study is to understand how natural selection is shaped by a combination of ecological and genetic constraints. More precisely, we investigate how the evolution of sex-specific investment in immune defences is affected by a combination of life-history trade-offs and pre-existing differences between male and female phenotypes. This enables us to revisit the general question asked by Stoehr & Kokko (2006): under what conditions should males and females evolve different levels of immunity? While sexual selection was an important component of their paper, we focus the present study on natural selection in order to understand how this process alone can lead to sex-specific evolution. Crucially, the evolutionary response of each sex depends on the phenotype of its counterparts as well as on pathogen dynamics, two factors that were missing from the previous model. To our knowledge, this is the first time that sexual reproduction has been incorporated in a model for the evolution of quantitative defences of hosts against pathogens. The framework presented here should be applicable to a wide range of evolutionary questions. In particular, sexual selection will be incorporated in this framework in a further study, which will enable us to gain a better understanding of the interactions between these two processes.

## 2. The model

### (a) Ecological and genetic framework

**Figure 1.**
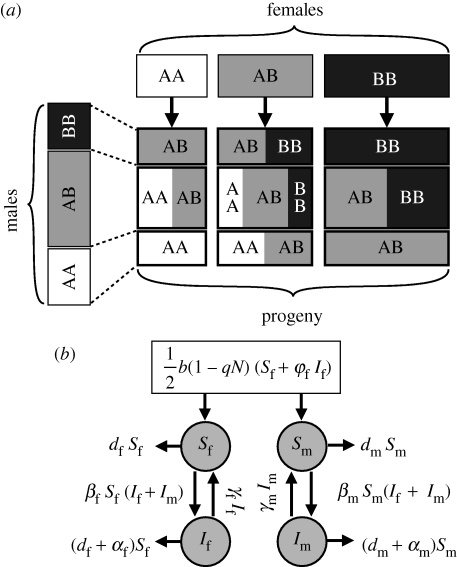
Schematic view of the main features of the model. (*a*) Sexual reproduction. This diagram represents the density (proportional to the size of the boxes) of individuals (reproductive females at the top, reproductive males on the left and their progeny in the middle) from each of three genotypes (focusing on one diploid locus with alleles A and B). The total density of offspring depends on the reproductive success of females (and is limited by ecological factors), while the genetic composition of the progeny is governed by the proportion of the genotypes in both males and females (Mendelian inheritance). (*b*) Population dynamics (ignoring genetic diversity). The discs represent susceptible and infected females and males (see table 1 for a list of symbols) and the arrows indicate flows of individuals out of or into the four compartments with their symbolic rates; the box at the top represents the birth rate, which is split evenly between males and females.

We consider a host species with two diploid sexes—female and male. Intraspecific competition for resources is modelled as a density-dependent birth rate reduction by a factor *qN*, where *N* is the total population density and *q* is a scaling factor assumed to be sex- and genotype-independent. Birth rate is proportional to the density of females. Following Bateman's (1948) principle, males compete for access to females, so that the reproductive success of male genotype *k* is proportional to its relative frequency (figure 1*a*). Note that we assume an unlimited supply of male gametes, so in theory a single male can mate with all the females in the population. Besides, there is no sexual selection in this model: mating between genotypes is homogeneous. Infected females *I*_f_ have a relative fecundity *φ*_f_ compared with uninfected females *S*_f_; likewise infected males *I*_m_ have a relative reproductive success *φ*_m_ compared with uninfected males *S*_m_. The birth rate of genotype *i* (equally split between males and females) is therefore given by
2.1
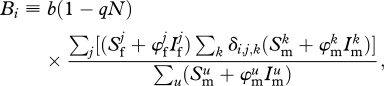

where superscripts (*j*, *k* and *u*) indicate genotype and *δ*_*i,j,k*_ represents the proportion of offspring with genotype *i* from a mother with genotype *j* and a father with genotype *k*, assuming Mendelian inheritance (figure 1*a*). For example, considering one locus with two alleles, A and B, the progeny of two heterozygous parents are in the following proportions: *δ*_AA,AB,AB_ = 1/4, *δ*_AB,AB,AB_ = 1/2, *δ*_BB,AB,AB_ = 1/4. In the full genetic model with two loci, we assume that there is no linkage disequilibrium, allowing free recombination.

This genetic model is then embedded in a classical susceptible–infected–susceptible model for pathogen transmission with no acquired immunity (figure 1*b*):
2.2
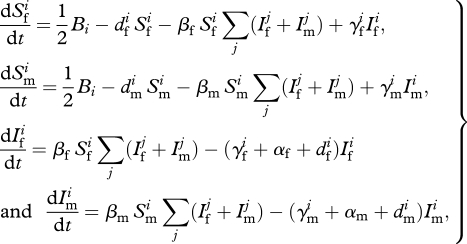

where subscripts f and m represent females and males, respectively, and *i* and *j* are placeholders for genotypes. Infectivity is independent of sex and genotype. We assume 1 : 1 sex ratio at birth. Traits that can vary with sex and genotype are the death rate (*d*), recovery rate (*γ*) and relative fecundity during infection (*φ*). Susceptibility to infection (*β*) and disease-induced mortality (*α*) are sex-dependent but genotype-independent within each sex. Table 1 lists the definitions and default numerical values of all the parameters used in this model. In the electronic supplementary material, appendix S1, we derive the expressions of the carrying capacity of the host and the basic reproductive ratio of the pathogen.

**Table 1.** Symbols used in the models and default numerical values of parameters (with arbitrary units).

symbol	definition	default value
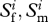	density of susceptible females/males with genotype *i*	n.a.
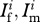	density of infected females/males with genotype *i*	n.a.
*B*_*i*_	birth rate of offspring with genotype *i* (as defined by equation (2.1))	n.a.
*N*	total host density in the population	n.a.
*δ*_*i*__.__*j,k*_	proportion of offspring with genotype *i* from mothers with genotype *j* and fathers with genotype *k*	n.a.
*b*	maximum fecundity of females	1
*q*	density-dependent reduction in fecundity	0.01
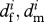	natural death rate of females/males with genotype *i*	0.25
*β*_f_, *β*_m_	infection rate of females/males	0.2
*α*_f_, *α*_m_	virulence (disease-induced death rate) in females/males	0.75
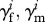	recovery rate of females/males with genotype *i*	2
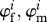	reproductive success of females/males with genotype *i* relative to non-infected individuals	1
*x*_f_, *x*_m_	proxy quantitative traits in females/males, determining the values of other traits of interest (equations (2.3*a*), (2.3*b*) and (2.3*c*))	n.a.

### (b) Genotype-to-phenotype mapping

We consider two diploid loci that are present in both sexes. The key assumption is that one locus controls the phenotype of females and the other controls that of males. In other words, the two loci code for sex-specific regulatory genes that affect the level of expression of other genes involved in immunity and life history. In the model, the pair of alleles at the first locus determines the value *x*_f_ of a dummy trait that affects both immunity and life history in females only. Similarly, the allele composition at the second locus determines the value of a trait *x*_m_ that affects immunity and life history in males only. In the following, we use the terms ‘male locus’ and ‘female locus’ with reference to their sex-specific phenotypic expression even though both loci are carried by both sexes.

We use pairwise-invasion plots (Boots & Haraguchi 1999) to explore the effect on host evolution of a range of trade-off functions (see complete list with exemplary plots in electronic supplementary material, appendix S2). In the main text, we focus on the specific functions below. As explained in that appendix and in §2*c*, these functions raise evolutionarily stable strategies (ESSs) over a wide range of parameter values, which allows us to explore quantitatively the selective pressures exerted on females and males in an extensive way. As shown in electronic supplementary material, appendix S2, other combinations of functions showed similar patterns.

We analyse three models that differ in the particular pairs of traits controlled by the values of *x*_f_ in females and *x*_m_ in males, assuming that both sexes obey identical trade-off functions (see electronic supplementary material, appendix S6, for sex-specific trade-off functions):

- recovery rate *γ* and background death rate *d* (with a positive relation),
2.3a
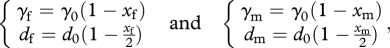

where *γ*_0_ and *d*_0_ are baseline parameter values;
- relative fecundity during infection *φ* and background death rate *d* (with a positive relation),
2.3b
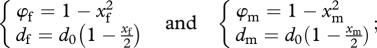

- recovery rate *γ* and relative fecundity during infection *φ* (with a negative relation),
2.3c
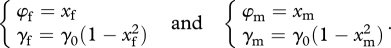

Following the terminology used by Boots *et al*. (2009), variation in recovery rate represents a form of resistance (i.e. a defence that reduces the reproductive success of the pathogen) while variation in fecundity during infection represents a form of tolerance (i.e. a defence that restores the host's fitness without affecting the pathogen's). In models (i) and (ii), both types of defence have a constitutive cost, expressed as a reduction in lifespan that is independent of infection. Model (iii) represents a resistance–tolerance trade-off, although tolerance can also be seen as a facultative cost of resistance, namely a reduction in reproductive success during infection. Alleles are assumed to have additive effects on traits *x*_f_ and *x*_m_ (NB: this does not imply additive effects on fitness). Thus, the phenotype of a heterozygote is the arithmetic mean of the two related homozygotes. Quantitative variation in sex-specific phenotypes is assumed to arise from infinite allele variation at a single locus, so that any value of *x*_f_ and *x*_m_ can be obtained by mutation.

### (c) Evolutionary stable strategies

We focus on ESSs (Geritz *et al*. 1997) as a way to compare the relative selective pressures applied to females and males. The complexity of our population dynamic model precludes the analytical derivation of the fitness function for a new, rare mutant genotype. Instead, we compute numerically the eigenvalues of system (2.2) when a homozygote population is at equilibrium and a new allele is introduced at very low frequency. A positive dominant eigenvalue indicates that the new allele will spread. It is therefore possible to determine numerically the existence of an ESS as follows. Consider a population in which locus 1 carries only allele A and locus 2 carries only allele B. We explore pairs of mutant alleles and determine the pair, A* and B*, that yields the largest dominant eigenvalue. We then systematically vary the original alleles, A and B, and repeat the process to find the condition under which A* = A and B* = B. We deem this to be an ESS because no other allele can persist, even in heterozygote individuals. Assuming that there is an infinite variety of alleles for each locus, we wrote an algorithm that searches the bi-dimensional space {0 < *x*_f_ < 1, 0 < *x*_m_ < 1} for an ESS, using Mathematica 7.0 (Wolfram Research 2008). In the electronic supplementary material, we provide more detail on the algorithm (electronic supplementary material, appendices S2 and S3); the source code is available upon request from the corresponding author.

## 3. Results

Our objective was to investigate how the two sexes respond evolutionarily to extrinsic changes in various parameters, given specific trade-off functions. The only qualitative difference between the sexes that was built in the model concerns the effect of intra-sex competition on reproductive success: males are competing for access to females, whereas females are competing for resources (equation (2.1)). Quantitative differences can then be introduced ad libitum, by varying the values of non-evolving traits, such as transmission rate or virulence, in males and females.

### (a) No extrinsic sex differences

**Figure 2.**
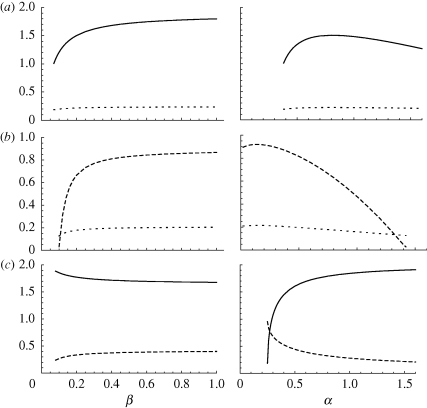
Evolutionarily stable strategies (ESSs) when males and females are equally exposed to and affected by infection. Curves show the different trait values at the ESS plotted against rate of infection *β* (left panels) or virulence *α* (right panel). (*a*) Model (i): trade-off between recovery rate *γ* (solid lines) and death rate *d* (dotted lines). (*b*) Model (ii): trade-off between relative reproductive success during infection *φ* (dashed lines) and death rate *d* (dotted lines). (*c*) Model (iii): trade-off between recovery rate *γ* (solid lines) and relative reproductive success during infection *φ* (dashed lines).

When all non-evolving traits are set equal in males and females, the two sexes evolve to identical ESSs (figure 2). If we increase simultaneously the susceptibility to infection of both sexes (*β*_f_ and *β*_m_), thus favouring the spread of infection, we obtain an increase in the ES investment in resistance (model (i)) or tolerance (models (ii) and (iii)). Note that with model (iii), higher transmission rates favour tolerance over resistance, in agreement with the asexual model (Restif & Koella 2004). Contrasting models (i) and (iii), we see that an increase in susceptibility (*β*) has opposite effects on the evolution of resistance (*γ*) depending on whether the cost is constitutive (model (i), increase in resistance) or facultative (model (iii), decrease in resistance). In the latter case, higher infectivity makes infection more likely to happen, thus increasing the effective cost of resistance.

When virulence (*α*) is increased in both sexes (figure 2*c*,*d*), the result is non-monotonic variation in ES investments in resistance (model (i)) or tolerance (model (ii)) when traded off against survival, in line with the asexual model (Restif & Koella 2004). Increasing virulence makes infection more costly to individual host survival, eventually leading to lower prevalence of infection in the population, which in turn reduces the effective benefits of host defences. This ecological feedback does not affect model (iii), where the cost of resistance is facultative. Indeed, with high virulence, hosts are less likely to be infected, but those who do get infected are very likely to die. So, provided that the cost of resistance is only apparent during infection, there is no incentive to evolve lower resistance as virulence increases. These results provide us with a basis for the study of sex-specific evolution of host defences.

### (b) Extrinsic sex differences

**Figure 3.**
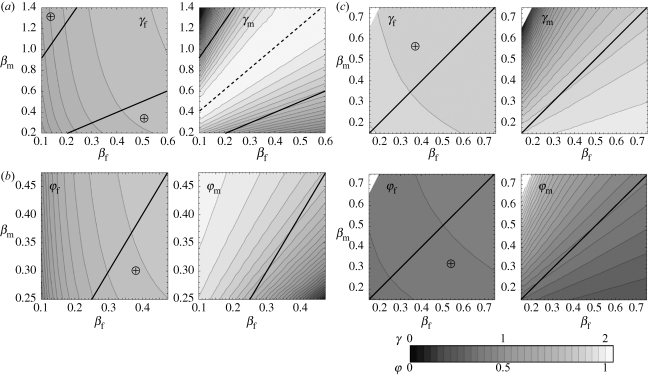
ESS variations with sex-specific infection rates *β*_f_ (horizontal axes) and *β*_m_ (vertical axes). Lighter shades represent higher values of the trait at the ESS. Circled crosses (⊕) indicate regions (delimited by solid lines) where females evolve higher immunocompetence than males. (*a*) Model (i), variations in female (left panel) and male (right panel) recovery rates at the ESS; the dashed line shows where the prevalence of infection in males at the ESS is 50%; note the different scales on the horizontal and vertical axes. (*b*) Model (ii), variations in female (left panel) and male (right panel) relative fecundity during infection at the ESS. (*c*) Model (iii), variations in female (left panels) and male (right panels) traits at the ESS: recovery rate in the upper row and relative fecundity during infection in the lower row.

We now introduce extrinsic differences between the sexes in some non-evolving traits, starting with susceptibility to infection (*β*_m_ and *β*_f_). As shown in figure 3, as soon as the susceptibilities of males and females differ, the two sexes evolve different investments in defence. Furthermore, males and females exhibit qualitatively different adaptive responses to extrinsic variations in susceptibility. Generally, male traits exhibit larger sensitivity to extrinsic changes than female traits, which is a consequence of the mating behaviour assumed in our model: competition is more intense among males than among females. Overall, higher resistance (model (i)) or higher tolerance (model (ii)) is selected for in females when the susceptibility of males or females increases, in agreement with the previous section. With model (iii), however, females evolve lower resistance when susceptibility increases in either sex because the effective cost of resistance increases following a raise in female prevalence (electronic supplementary material, appendix S7).

Male evolution shows more complicated patterns (figure 3), but these can be understood by observing the effects of changes in sex-specific susceptibility on infection prevalence in males (electronic supplementary material, appendix S7). Increasing female susceptibility causes a drop in prevalence among males, because disease-induced mortality in females induces of a decline in population size, hindering pathogen transmission. Unsurprisingly, this selects for lower male resistance (model (i), provided that male susceptibility is not too high) or lower male tolerance (model (ii)) when the cost of defence is constitutive. With model (iii), the decrease in prevalence lowers the effective cost of resistance; hence there is a positive response of the ES level of resistance in males to changes in female susceptibility. In contrast, higher male susceptibility results in increased prevalence among males (electronic supplementary material, appendix S7). This selects for higher male tolerance with model (ii), lower male resistance when the cost is facultative (model (iii)) and higher male resistance when the cost is constitutive (model (i)) as long as male prevalence remains below 50 per cent (i.e. below the dashed line in figure 3*a*). When the prevalence of infection among males exceeds 50 per cent, any further increase in male susceptibility or decrease in female susceptibility selects for reduced investment in male resistance with model (i). This is because male hosts are rapidly reinfected after they recover, so the effective benefits of immunity drop below its cost.

An unexpected consequence of the non-monotonic response of males to changes in sex-specific susceptibility with model (i) is the prediction that males can evolve a lower recovery rate than females when male susceptibility is much higher than female susceptibility (figure 3*a*). In contrast, when the cost of resistance is facultative (model (iii)), the sex with higher susceptibility (or exposure) to infection always evolves lower resistance than its counterpart (figure 3*c*). This is in order to counteract the higher effective cost of immunity. Note the convergence of evolutionary patterns in males with models (i) and (iii) when prevalence is high: if males are regularly reinfected, there is little difference between a facultative and a constitutive cost of immunity.

**Figure 4.**
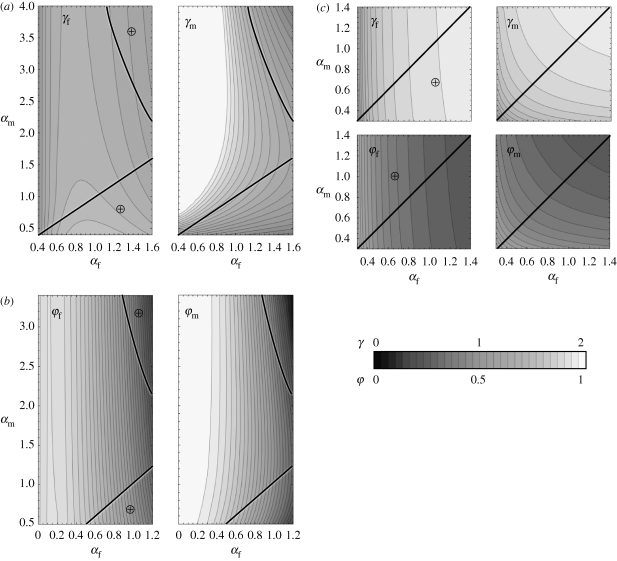
The effect of the ESS in the two sexes for different values of pathogen virulence in females *α*_f_ (horizontal axes) and in males *α*_m_ (horizontal axis). Diagrammatic representation of data is as for figure 3; see figure 3 legend for explanation. (*a*) Model (i), (*b*) model (ii) and (*c*) model (iii).

Extrinsic sex-specific variation in virulence (i.e. infection-induced mortality) does not create a similar asymmetry between male and female evolution. With either model (i) or (ii), where the cost of defence is constitutive, an increase in virulence in one sex results in a non-monotonic evolutionary response in that sex (the ES investment in immunity reaching a maximum at an intermediate level of virulence), and in a steady decrease in immune defences in the other sex (figure 4). This is in agreement with the explanation put forward in the previous section: at the individual level, higher virulence represents an increased cost of infection, while at the population level, higher virulence in either sex leads to a decreased risk of getting infected for both sexes. Accordingly, the latter effect does not affect host evolution in model (iii) since both the benefit and the cost of immunity are facultative. So, overall, males and females exhibit similar evolutionary responses to extrinsic changes in sex-specific virulence. Quantitatively, selective pressure is generally less sensitive to changes in male-specific virulence than changes in female-specific virulence (figure 4). This is because changes in female-specific virulence have a much stronger effect on demography than changes in male-specific virulence (results not shown). As a consequence of the non-monotonic responses in both sexes when the cost of defence is constitutive (model (i) or (ii)), high levels of virulence can select for higher investment in female-specific defences than in male-specific defences when virulence in males exceeds that in females (and *vice versa*). This reversal does not reflect patterns of sex-specific prevalence (the sex suffering higher virulence has a lower prevalence), but rather the fact that the male ESS is more sensitive to changes in virulence than the female ESS.

## 4. Discussion

Our results show that, under a range of genetic and ecological constraints, males and females can evolve different levels of immune defences, sometimes at odds with intuitive expectations. Even though specific patterns are likely to vary among species, we have identified some of the key factors that should be taken into account to understand the selective pressures involved.

### (a) Contrasting male and female strategies

The first question to consider is what we mean by ‘male’ and ‘female’, in the sense of characteristics that create specific selective constraints on the evolution of other traits. We used as a starting point Bateman's (1948) seminal observation that males tend to experience stronger intra-sexual competition than females, expressed by Zuk (2008) as a male strategy to ‘live hard, die young’, and hence perhaps to invest less in immunity, though the generality of Bateman's principle has been widely debated (Clutton-Brock & McAuliffe 2009). We found that Bateman's principle in itself does not select for different investments in immunity in the two sexes, provided that (i) they are equally exposed to and affected by infection and (ii) they undergo identical genetic constraints. This agrees with Stoehr & Kokko's (2006) initial results in the absence of sexual selection. In this study, we focused on the effects of releasing the former assumption. Releasing the latter assumption (i.e. identical genetic constraints) has a straightforward effect: the sex with the lower built-in cost evolves stronger immunity (electronic supplementary material, appendix S6). Although there is evidence for sex-specific differences in the physiological cost of immunocompetence (Leman *et al*. 2009; Malo *et al*. 2009), we could not find quantitative information on the trade-offs involved.

We assessed the selective pressures on a subset of sex-specific traits (recovery rate, reproductive success during infection and lifespan) caused by arbitrary differences between males and females in infection rate or virulence (i.e. disease-induced death rate). In so doing, we covered a range of scenarios whereby sex-specific reproductive traits such as hormones and behaviour could plausibly affect the exposure to infection (Semple *et al*. 2002; Grear *et al*. 2009) or the severity of disease (Cernetich *et al*. 2006). First, we showed that changes in the traits of either sex affect the selective pressures on both sexes, either in the same or in opposite directions, depending on the ecological feedbacks. For example, an increase in male susceptibility (or exposure) to infection favours the spread of the pathogen in the whole population and therefore tends to select for higher resistance or tolerance in both sexes if the cost of immunity is constitutive. However, above a certain level of exposure, the benefit of rapid recovery in males decreases owing to constant reinfection (we assume no acquired immunity). This selects for lower resistance in males, ultimately leading to the counterintuitive situation where males with higher susceptibility or exposure to infection than females evolve lower immunocompetence (figure 3). A similar pattern arises if the cost of immunity is facultative, in the form of a trade-off between rate of recovery and relative fecundity during infection (model (iii)): if males happen to be more susceptible (or exposed) to infection than females, they are predicted to evolve a longer infectious period balanced by higher sexual activity during infection than females.

### (b) Ecology and immunity

Beyond the specific predictions made above, which may be difficult to validate empirically without detailed information on the trade-offs involved in any particular species, our study highlights the importance of ecological feedbacks on adaptive dynamics. In order to make predictions about how selective pressures drive the evolution of a system, it is necessary to understand both the genetic or physiological constraints faced by an individual and the impact that any change in life-history traits in the population has on the immediate environment (Mylius & Diekmann 1995). Traditional models of life-history evolution (following Maynard Smith 1979) only account for direct competitive interactions in order to evaluate the fitness of a mutant genotype in a population. However, when dealing with the evolution of immune defences, it is essential to take into account the effect of immunity on host–pathogen dynamics. This was emphasized in previous studies where forms of defence that lead to a decrease in pathogen prevalence could be counterselected because of a negative epidemiological feedback (Boots & Haraguchi 1999; Roy & Kirchner 2000; Restif & Koella 2004).

Evolutionary models such as Stoehr & Kokko's (2006) that ignore host–pathogen dynamics miss an important element when they assess the benefits of immunity, because these are not fixed. Indeed, the benefit of immunity is determined by both the probability of infection, itself a function of prevalence of infection in the population and individual susceptibility or exposure, and the fitness cost of being infected owing to reduced survival or fecundity. For example, a more infectious pathogen will increase the probability of infection, whereas a more virulent pathogen will have two opposing effects: an increase in the individual cost of infection and a decrease in the probability of infection (if early death reduces the infectious period). In a clonal host species, higher infectivity selects for increased investment in defences and ultimately higher tolerance than resistance, whereas higher virulence selects for decreased investment in defences and ultimately higher resistance than tolerance (Restif & Koella 2004). Extending these models to a sexual species shows that these patterns are preserved, but only when both sexes are equally affected by infection (figure 2), otherwise the sexes evolve different rates of recovery or virulence (figures 3 and 4).

The main reason why the sexes evolve differently with respect to infection appears to come from the way in which extrinsic changes in parameters affect ecological dynamics, and therefore the probability of infection of each sex. We assumed that infection is density-dependent and that population density is driven by female fecundity, and this leads to female and male traits having asymmetric effects on sex-specific prevalence (electronic supplementary material, appendix S7). Thus, when we also allowed for both the benefit and the cost of immunity to be condition-dependent or facultative (model (iii)), in a direct resistance–tolerance trade-off, while female trait evolution followed Restif & Koella's (2004) predictions, male resistance was unexpectedly found to increase in response to higher female susceptibility. Again, this was due to the decrease in male prevalence resulting from the negative impact of female infection on population density. Naturally, the exact behaviour of our models depends on specific assumptions about demography, epidemiology and genetics, and these will vary between systems. However, our key message is that all these factors interact to determine the direction and strength of sex-specific selective pressures. To ignore any one component or link may result in misleading conclusions.

### (c) Further implications

One of the main challenges in designing this model was the need for an explicit genotype-to-phenotype map to account for sexual reproduction and sex-specific quantitative traits. In the absence of empirical information on the genetic determinism of sex differences in immunity, we had to make a number of simplifying assumptions in order to ensure that the model remained tractable. Despite this, we have developed a novel way of incorporating sex into classical adaptive dynamic models designed to explore asexual host species evolution. We believe our framework will prove both versatile and flexible enough to be used in a range of future studies on sexual host species.

Our approach was to explore the behaviour of the model by assuming that certain traits are fixed while others can evolve under particular genetic constraints. This is of course an artificial situation because one would expect all traits to have the potential to evolve. Our objective here was to illustrate the complex interactions between genetics and ecology that are likely to occur in nature by focusing on a small number of relatively simple scenarios. Following Restif & Koella (2003), a natural extension would be to assume that the rate of infection or the rate of infection-induced death (virulence) actually depends on the combination of host and pathogen genotypes. Such genotype-by-genotype interactions have been demonstrated in natural systems (Salvaudon *et al*. 2008), and there are no reasons why the sex of the host may not play a role too.

While we have focused on an infection transmitted by direct contact between any pair of hosts, there are at least two other situations where host sex is known to play a major role in infection dynamics: sexually transmitted infections and vertical transmission from mother to offspring. A further aspect that we have deliberately ignored is pathogen evolution, which would add an additional layer of complexity to the model (van Baalen 1998; Restif & Koella 2003; Best *et al*. 2009). The effect of host sex dimorphism on pathogen dynamics (Adler *et al*. 2008) and evolution (Fellous & Koella 2009) has only recently started to be documented. Some insight into how pathogens may respond to a range of different phenotypes between sexes might be gained by extending previous models that accounted for other forms of host diversity (Green *et al*. 2006; Fraser *et al*. 2007). Indications are that host heterogeneity generally reduces pathogen spread, and sex-specific immunity profiles could help to strengthen this effect.

Finally, another important extension of this model will be to consider the effect of sexual selection on host evolution. Stoehr & Kokko (2006) predicted that female choosiness would select for lower immunocompetence in males, which could be tested when population dynamics and genetics are accounted for. Our modelling framework should allow the study of mate choice evolution in the presence of an infectious pathogen, thus adding an ecological dimension to recent life-history (Adamo & Spiteri 2005, 2009; Kokko *et al*. 2006) and population-genetic models (Howard & Lively 2003). Related to this issue is the effect of interspecific mating strategy variation (e.g. monogamy versus polygamy), which Zuk (1990) predicted to be a major determinant in sex-specific evolution. We hope to provide a functional modelling framework that will enable more specific predictions to be formulated and tested empirically.
